# Effect of MDM2 SNP309 and p53 codon 72 polymorphisms on lung cancer risk and survival among non-smoking Chinese women in Singapore

**DOI:** 10.1186/1471-2407-10-88

**Published:** 2010-03-10

**Authors:** Hui Wan Chua, Daniel Ng, Serena Choo, San San Lum, Huihua Li, Li Ying Soh, Kanaga Sabapathy, Adeline Seow

**Affiliations:** 1 Division of Cellular & Molecular Research, Humphrey Oei Institute of Cancer Research, National Cancer Centre, 11, Hospital Drive, Singapore 169610, Singapore; 2 Department of Epidemiology and Public Health, Yong Loo Lin School of Medicine, National University of Singapore, Singapore 117597, Singapore; 3 Division of Clinical Trials and Epidemiological Sciences, Humphrey Oei Institute of Cancer Research, National Cancer Centre, 11, Hospital Drive, Singapore 169610, Singapore; 4 Dept of Biochemistry, Yong Loo Lin School of Medicine, National University of Singapore, Singapore 117597, Singapore; 5 Cancer and Stem Cell Biology Program, Duke-NUS Graduate Medical School, 2 Jalan Bukit Merah, Singapore 169547, Singapore

## Abstract

**Background:**

Single nucleotide polymorphism (SNP) 309 resulting in a T or G allele in the promoter of *MDM2*, the negative regulator of p53, has been suggested to affect cancer predisposition and age of onset, primarily in females. However, findings have been inconsistent in various cancers, and ethnicity appears to be a critical factor influencing the effects of the SNP on cancer risk. An increasing trend has been observed in the prevalence of lung cancers in non-smokers, especially females, though the underlying genetic basis is unclear.

**Methods:**

We therefore examined the role of the SNPs in the p53 pathway (*p53 *codon 72 and *MDM2 *SNP309) on lung cancer risk and prognosis of a life-time non-smoking female Chinese population, in a hospital-based case-control study of 123 cases and 159 age-matched controls, by PCR analysis.

**Results:**

Our findings reveal that the risk of lung cancer among individuals with the *MDM2 *SNP309 TT genotype was 2.1 (95% CI 1.01-4.36) relative to the GG genotype, contrary to initial expectations that the GG genotype with elevated MDM2 levels will increase cancer risk. Those who had this genotype in combination with the *p53 *Pro allele had a risk of 2.5 (95% CI 1.2-5.0). There was however no effect of either polymorphism on age at diagnosis of lung cancer or on overall survival.

**Conclusions:**

The results thus demonstrate that the MDM2 SNP309 TT rather than the GG genotype is associated with increased risk of lung cancer in this population, suggesting that other mechanisms independent of increased MDM2 levels can influence cancer susceptibility.

## Background

The TP53 tumour suppressor pathway plays a critical role in cell cycle regulation and apoptosis in many cancers, including lung carcinomas [1], and variation in the genes that regulate this pathway may exert an important influence on tumour development, and hence, cancer risk. Recent interest has focused on the murine double minute-2 protein (MDM2), a nuclear phospoprotein that inhibits p53 activity by promoting its degradation [2]. A single nucleotide polymorphism (SNP309) in the *MDM2 *promoter has been found to influence transcription of this gene via a greater affinity for the SP1 transcription factor, and hence, individuals with the GG genotype have higher MDM2 levels leading to attenuation of the p53 pathway [3,4]. This has been especially so in the case of females, due to the involvement of the MDM2 SNP in the estrogen receptor signaling pathway [3].

Several epidemiologic studies have evaluated this association with varying results. No overall association between *MDM2 *SNP309 and lung cancer risk was observed in recent studies in a European [5], North American [6,7] and two Asian [8,9] populations. However, the GG genotype was observed to increase risk in a study in China [10], Norway [11] and among non-Hispanic Whites in the US [12]. In some cases, even though there was no overall association noted with the *MDM2 *SNP309 and cancer susceptibility, substratification of groups led to some association. For example, an increased risk was correlated with the GG genotype only in the adenocarcinomas, but not in the small cell, squamous cell or non-small cell lung cancers (NSCLCs), or when all lung cancer subtypes were grouped [9]. Other studies pointed to an interaction with smoking status - although no association was found between all lung cancers and the *MDM2 *SNP309, there was an elevated risk of cancer susceptibility in smokers compared to non-smokers in the same study [10]. Conversely, one study found that the risk of lung cancer was higher among individuals with the TT genotype - on stratification, this association was restricted to males and to smokers [12]. A meta-analysis of seven studies concluded that the GG genotype conferred a small but significant increased risk (summary odds ratio [OR] 1.27; 95% confidence interval [CI] 1.12-1.44) [13]. Because of the capacity of the polymorphism to enhance the inhibitory action of MDM2 on the p53 pathway, investigators have hypothesized that the GG genotype would also negatively influence prognosis in lung cancer. To date, two studies have demonstrated that the GG genotype is associated with a lower level of p53 expression [14] and with poorer survival [14,15].

A polymorphism at codon 72 of the *p53 *gene resulting in an arginine-to-proline (Arg/Pro respectively) substitution has also been shown to be varyingly associated with cancer predisposition, possibly due to differences in ethnicity and in background risk due to smoking [16-18]. A meta-analysis of 13 epidemiologic studies which examined this association reported that the summary odds ratio of lung cancer associated with the Pro/Pro genotype was 1.18 (95% CI 0.99-1.41), and was 1.02 (95% CI 0.86-1.20) for Pro-carriers [19]. The Pro/Pro genotype has also been associated with poorer prognosis and less favorable clinical outcome in lung cancer patients [14,20,21]. Moreover, significantly higher or a lower prevalence of *p53 *gene mutations have been noted in lung cancers among carriers of the Pro allele in the Polish [22] or a Norwegian population [23], respectively. These data highlight that ethnicity and other factors may have an impact on p53 functionality, with respect to the SNP and mutational status.

The influence of gender and ethnic differences on genetic determinants of lung cancer is increasingly being recognized. Smoking accounts for the overwhelming majority of cases in Western populations and among men, but lung cancer among non-smokers is now known to be a distinct clinico-pathologic entity that is as yet poorly understood [24,25]. We therefore sought to examine if MDM2, through its role in the p53 pathway, is associated with risk and prognosis of lung cancer among Singapore Chinese women, a primarily non-smoking population. In addition, as *MDM2 *SNP309 status has been demonstrated to affect the overall age of onset of various cancers, with differential effects in different populations [4,26], we have explored its effects in this unique population.

## Methods

### Study population

Participants were drawn from a hospital-based case-control study on lung cancer among Chinese women in Singapore [27]. Incident cases were primary lung cancers diagnosed at any one of three major hospitals in the country over the study period, and controls were patients admitted to the same hospitals, frequency-matched by age. Patients admitted for malignant or chronic respiratory conditions (chronic bronchitis, emphysema and chronic asthma) were not eligible to participate as controls. All participants provided written consent. Within this study population, a consecutive sub-sample of participants were asked, and consented to provide a blood specimen for genetic analysis. Of these, 126 cases (95.2% of which were pathologically confirmed) and 162 controls were never smokers and form the basis for the current report. The study was approved by the Ethics Committee of the National Cancer Centre and the Institutional Review Board of the National University of Singapore.

Demographic, smoking and other relevant information was obtained by in-person interview with a trained nurse. A lifetime non-smoker was defined by a negative response to the question "Have you ever smoked a cigarette or any other form of tobacco, at least once a day for one year?"

Genomic DNA was prepared from peripheral blood using the standard proteinase K-phenol-chloroform procedure and DNA was stored at -30°C till analysis.

### Laboratory analysis

Genotyping was performed independently at the Department of Community, Occupational and Family Medicine, National University of Singapore, and at the Laboratory of Molecular Carcinogenesis, National Cancer Centre. Genomic DNA from peripheral blood samples was used for PCR analysis of *p53 *using the following primers: Forward: 5'-GAAGACCCAGGTCCAGATGA-3' and Reverse: 5'-ACTGACCGTGCAAGTCACAG-3', this gave rise to a 216 bp product, followed by BtgI digestion. The *p53 *Pro allele has a unique *BstUI *site that is absent in the Arg allele, resulting in bands of different sizes as follow: 50 bp, 166 bp (Pro/Pro), 216 bp (Arg/Arg) and 50 bp, 166 bp, 216 bp (Pro/Arg). Likewise, *MDM2 *promoter SNP309 was amplified as a 194 bp product using the following primers: Forward: 5'-CGGGAGTTCAGGGTAAAGG-3' and Reverse: 5'-TCGGAACGTGTCTGAACTTG-3'. Genotyping was then performed by digestion using restriction enzyme MspAI. The MDM2 promoter SNP309 G allele has a unique MspAI site that is absent in the T allele resulting in bands of different sizes as follows: 49 bp, 145 bp (G/G), 194 bp (T/T), 49 bp, 145 bp, 194 bp (G/T). MDM2 status was also confirmed by sequencing reactions using the following primers: Forward: 5'-CGGGAGTTCAGGGTAAAGGT-3' and Reverse: 5'-AGCAAGTCGGTGCTTACCTG-3', as described [26].

### Statistical analysis

We used unconditional logistic regression to compute adjusted odds ratios (ORs) and their corresponding 95% confidence intervals (CIs) for the association between lung cancer risk and *MDM2 *SNP309 and *p53 *codon 72 genotypes. ORs were adjusted for age (in years) and birthplace. All participants were female, never smokers and ethnic Chinese.

The Kruskal-Wallis test was carried out to evaluate the differences in the age of onset of cancer among patients with different genotypes, while Fisher's exact test was used to test the equivalence of distribution of cancer stage among patients with different genotypes. The Logrank test was performed to examine the differences between Kaplan-Meier-estimated overall survival among patients with different genotypes. Analyses were performed using STATA version 9.0 (Strata Corporation, College Station, TX USA) and SPSS for Windows version 15.0 (SPSS Inc., Chicago, IL).

## Results

Table 1 provides a description of relevant demographic and other background characteristics of cases and controls. They were similar in respect of age, years of education, and cases were more likely to have been born outside of Singapore than controls. They were also less likely to consume fruit and vegetables, or to be exposed to environmental tobacco smoke at home. Approximately three-quarters of the cases were adenocarcinomas.

**Table 1:** Distribution of selected characteristics among cases and controls^1^

	Cases (n, %)	Controls (n, %)
Age in years^2 ^(Mean ± S.D.)	62.0 ± 13.7	63.4 ± 12.3
Years of formal education (Mean ± S.D.)	3.5 ± 4.2	3.8 ± 4.8
Servings of fruit weekly (median, IQR)	3.9, 6.8	7.8, 10.1
Servings of vegetable weekly (median, IQR)	18.1, 16.0	20.6, 20.1
Birthplace		
Singapore/Malaysia	82 (65.1)	130 (80.2)
China/other	44 (34.9)	32 (19.8)
Exposure to environmental tobacco smoke at home		
Daily	46 (36.5)	72 (44.4)
Weekly < daily	16 (12.7)	15 (9.3)
Less than weekly	64 (50.8)	75 (46.3)
Histological subtype		
Squamous/small cell carcinoma	14 (11.9)	
Adenocarcinoma	85 (72.0)	
Large cell undifferentiated/Not otherwise specified	19 (16.1)	

^1 ^All participants are Chinese women, cases and controls frequency-matched for age. A total of 126 cases and 162 controls were eligible, of which 123 and 159, respectively, were successfully genotyped.

^2 ^Refers to age at diagnosis (cases) and age at interview (controls)

A total of 123 (97.6%) cases and 159 (98.1%) controls were successfully genotyped for both polymorphisms. Genotype frequencies were comparable with the published literature and in keeping with the lower prevalence of the T allele reported in Asian, relative to Caucasian, populations [7,8,10-12]. The distribution of genotype frequencies conformed to that expected under the Hardy-Weinberg equilibrium for both MDM2 SNP309 (p = 0.62) and TP53 codon 72 (p = 0.19).

### MDM2 SNP309 TT genotype increases lung cancer risk

Risk of lung cancer was higher among individuals with the *MDM2 *SNP309 TT genotype relative to the GG genotype (OR 2.10, 95% CI 1.01-4.36) (Table 2). Heterozygotes showed an intermediate risk (OR 1.42, 95% CI 0.80-2.52).

**Table 2:** Risk of lung cancer in relation to MDM2 SNP309 and p53 codon 72 polymorphisms

Genotype		Cases (n = 123)	Controls (n = 159)	**Age- and birthplace-adjusted**^1^**odds ratio (95% CI)**
			
		No. (%)	No. (%)	
MDM2 SNP309				
GG		29 (23.6)	51 (32.1)	1.00
TG		65 (52.8)	83 (52.2)	1.42 (0.80-2.52)
TT		29 (23.6)	25 (15.7)	2.10 (1.01-4.36)
P53 codon 72				
Arg/Arg		28 (22.8)	42 (26.1)	1.00
Arg/Pro		69 (56.1)	88 (54.7)	1.21 (0.67-2.17)
Pro/Pro		26 (21.1)	31 (19.3)	1.44 (0.70-3.00)
Combined genotype^2^				
P53 codon 72	MDM2 SNP309			
Arg/Arg	GG/TG	24 (85.7%)	32 (76.2%)	1.00
	TT	4 (14.3%)	10 (23.8%)	0.58 (0.15-2.22)
ArgPro/ProPro	GG/TG	69 (73.4%)	102 (87.2%)	1.00
	TT	25 (26.6%)	15 (12.8%)	2.42 (1.16-5.03)

^1 ^Adjusted for age in years and birthplace (China-born, Singapore/Malaysia-born). All individuals were lifetime non-smoker Chinese women.

^2 ^P (interaction) = 0.06

Analysis of the *p53 *codon 72 SNP revealed that the presence of the *p53 *Pro allele was associated with a small but non-significant increase in risk: odds ratios for Arg/Pro and Pro/Pro genotypes were 1.21 (95% CI 0.67-2.17) and 1.44 (95% CI 0.70-3.00), respectively (Table 2).

We next evaluated if the effect of *MDM2 *SNP was modified by *p53 *codon 72 status. The TT genotype conferred an elevated risk (OR 2.42, 95% CI 1.16-5.03) only among codon 72 Pro allele carriers (Table 2). However, the interaction was not statistically significant (p = 0.06). A similar magnitude of risk (OR 2.47, 95% CI 1.21-5.03) (data not shown) was conferred by the TT, Arg/Pro, Pro/Pro genotypes in combination, relative to the GG, Arg/Arg genotypes.

Taken together, the data indicate that the TT genotype of *MDM2 *SNP309 is associated with increased lung cancer risk.

### p53 codon 72 polymorphism and MDM2 SNP309 status does not influence the age of diagnosis of lung cancer

**Figure 1:**
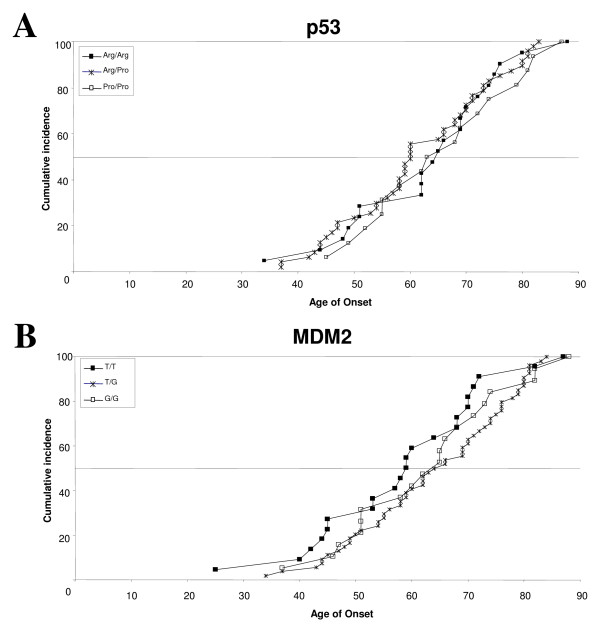
***MDM2 SNP309 *and *p53 *codon 72 polymorphism do not affect onset-age of lung cancer in female non-smoker patients**. **A-B**. The cumulative incidence of cancer cases in the population was plotted as a function of age of onset of lung cancer, comparing the various *p53 *codon 72 (**A**) and *MDM2 SNP309 *(**B**) genotypes. No significant differences were noted with respective to both the different *p53 *and *MDM2 *genotypes. n = 21 for Arg/Arg, n = 16 for Pro/Pro and n = 47 for Arg/Pro and n = 22 for T/T, n = 19 for G/G and n = 54 for G/T.

Kruskal-Wallis analysis indicated no association of the *p53 *codon 72 genotypes with age of onset in the study population (p = 0.557); 50% of the population were diagnosed between the ages of 60 and 63, regardless of the genotypes (Figure 1A), and the median age of onset for the Arg/Arg, Arg/Pro and Pro/Pro groups were found to be 64.5, 60.0 and 63.0, respectively. Similar analysis of the effect of the various *MDM2 *SNP309 genotypes also revealed no association between them and the age of onset of lung cancer (p = 0.338) (Figure 1B). The median age of onset for the T/T, T/G and G/G groups were found to be 59.0, 65.0 and 63.5, respectively (Figure 1B). Combined analysis of the effects of both *p53 *and *MDM2 *alleles also did not reveal any significant differences in the age of onset of the disease (p = 0.718)(data not shown).

### Overall survival is not influenced by p53 codon 72 polymorphism and MDM2 SNP309 SNP

**Figure 2:**
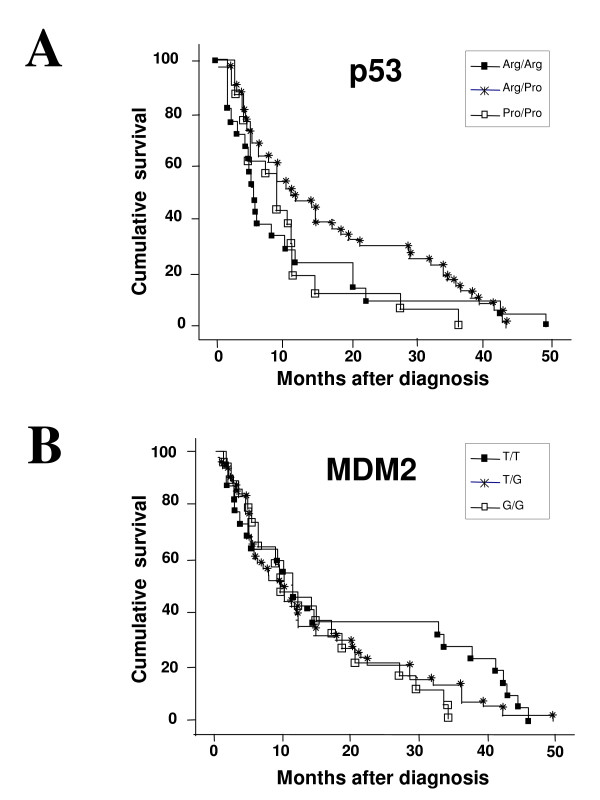
**Polymorphic status of p53 codon 72 or MDM2 SNP309 does not affect overall survival of female non-smoker lung cancer patients**. **A-B**. Kaplan-Meier overall survival curves for all patients with different p53 (A) or MDM2 (B) genotypes. No significant differences were noted with respective to the different MDM2 or p53 genotypes alone or in combination. n = 21 for Arg/Arg, n = 16 for Pro/Pro and n = 47 for Arg/Pro and n = 22 for T/T, n = 19 for G/G and n = 54 for G/T.

97 patients with survival data available were included in the survival analysis. The median follow-up time was 10.1 months. Analysis of overall survival revealed no statistically significant differences with respect to the various *p53 *genotypes (p = 0.263) (Figure 2A). The median survival for patients with Arg/Arg, Arg/Pro and Pro/Pro *p53 *genotypes were 5.47 (CI 95% 3.17-10.23), 10.87 (CI 95% 7.53-18.70) and 8.93 (CI 95% 4.33-11.63) months, respectively (Figure 2A). Similarly, no significant differences were observed with respect to the various *MDM2 *genotypes and overall survival (p = 0.267) (Figure 2B), and the median survivals for the various *MDM2 *SNP309 alleles were 11.17 (CI 95% 3.60-33.80) (T/T), 10.10 (CI 95% 5.47-11.90) (T/G) and 9.57 (CI 95% 5.17-18.70) (G/G) months (Figure 2B).

Combined analysis of the effects of both *p53 *and *MDM2 *polymorphisms on overall survival also did not reveal any significant differences among the nine different genotypes (p = 0.367) (data not shown).

## Discussion

In summary, we show here that the *MDM2 *SNP309 TT genotype is associated with a higher risk of lung cancer among never smoking women in the Chinese population. The presence of the *p53 *codon 72 Pro allele did not lead to a statistically significant interaction or materially affect the magnitude of this risk. Neither of the *MDM2 *nor *p53 *polymorphisms evaluated showed any impact on survival or age of onset of lung cancer in this population.

Our study population is unique in consisting solely of non-smoking women, who could potentially be informative with regard to risk factors that operate independently of tobacco smoking. The finding that the *MDM2 *TT genotype increases risk was unexpected, since as described earlier, many studies that have reported associations have found increased risk with the GG genotype that are consistent with the impact of this genotype on *MDM2 *RNA and protein levels, and hence, on inhibition of p53 [3]. To our knowledge, only one study has examined the combined effect of *MDM2 *and *p53 *polymorphisms and lung cancer, and this was also conducted in a Chinese population [10]. The results showed a multiplicative effect, with a 4.5-fold higher risk in those with the GG, Pro/Pro genotypes relative to TT, Arg/Arg. However, our findings suggesting that TT genotype can be a susceptibility factor are not entirely surprising, given the recent observations. Recent work using large numbers of breast cancer samples in the Chinese female population also revealed that the TT genotype predisposes to accelerated onset of cancers [26], and that the GG genotype reduced risk of leukemia [28]. Moreover, one other study has noted association of increased lung cancer risk with the TT genotype in the Chinese population [12], supporting our data. Thus, it is possible that the association of the G or the T alleles with increased cancer risk or onset age may be influenced by ethnicity and by environmental factors unique to that particular population. More in depth larger scale studies are now required to further extend these findings.

How does the TT genotype lead to increased cancer risk is at present not well understood, but there is evidence to suggest that the role of MDM2 in tumorigenesis may vary in a gender-specific manner, and between smokers and never smokers. Thus, it is not inconceivable that the mechanisms by which MDM2 modulates lung cancer risk can differ between populations. The role of hormones in carcinogenesis is rapidly emerging as a complex, but important pathway. Estrogen signaling, in particular the estrogen receptor (ER), is known to play a direct role in *MDM2 *transcription, regulating its expression [29]. The G allele of SNP309 increases the affinity of the *MDM2 *promoter for Sp1, a co-transcriptional activator for the estrogen receptor, and was associated with increased MDM2 expression. Consistently, Bond et. al. showed that the effect of the *MDM2 *polymorphism (GG) was gender-specific and enhanced in women with active estrogen signaling pathways [30]. Therefore, one would envisage that the TT genotype is associated with reduced MDM2 levels. However, MDM2 has also been shown to negatively regulate ER expression [31]. Thus, it is possible that reduced MDM2 levels expected in TT individuals could lead to elevated ER expression, and hence may elevate cancer risk - a hypothesis that requires further investigation. Given that estrogen receptors are expressed in lung tumours, particularly adenocarcinomas, and these cells are responsive to estrogen [32,33], it is possible that hormonal pathways provide an alternative mechanism by which MDM2 influences lung cancer risk in this population. Alternatively, other MDM2-dependent pathways could be modified in the TT subjects, leading to cancer susceptibility. In this respect, no studies have evaluated if specific signaling pathways are associated with the MDM2 SNPs. Given the emergence of an association of the TT genotype with cancer susceptibility in various studies, such analyses would provide further mechanistic insights.

## Conclusions

In conclusion, our findings suggest that the *MDM2 *SNP309 TT genotype is a risk factor for lung cancer in never-smoker Chinese females. The difference between our results and others highlights the possibility that MDM2 may operate via unique mechanisms among non-smoking Asian women.

## Competing interests

The authors declare that they have no competing interests.

## Authors' contributions

HWC, DN, SC, SSL and LYS carried out the molecular genetics studies. HL carried out statistical analysis. KS and AS designed the study, interpreted the data and wrote the manuscript. All authors read and approved the final manuscript.

## Pre-publication history

The pre-publication history for this paper can be accessed here:

http://www.biomedcentral.com/1471-2407/10/88/prepub
